# A Consistent Definition of Phase Resetting Using Hilbert Transform

**DOI:** 10.1155/2017/5865101

**Published:** 2017-05-03

**Authors:** Sorinel A. Oprisan

**Affiliations:** Department of Physics and Astronomy, College of Charleston, 66 George Street, Charleston, SC 29424, USA

## Abstract

A phase resetting curve (PRC) measures the transient change in the phase of a neural oscillator subject to an external perturbation. The PRC encapsulates the dynamical response of a neural oscillator and, as a result, it is often used for predicting phase-locked modes in neural networks. While phase is a fundamental concept, it has multiple definitions that may lead to contradictory results. We used the Hilbert Transform (HT) to define the phase of the membrane potential oscillations and HT amplitude to estimate the PRC of a single neural oscillator. We found that HT's amplitude and its corresponding instantaneous frequency are very sensitive to membrane potential perturbations. We also found that the phase shift of HT amplitude between the pre- and poststimulus cycles gives an accurate estimate of the PRC. Moreover, HT phase does not suffer from the shortcomings of voltage threshold or isochrone methods and, as a result, gives accurate and reliable estimations of phase resetting.

## 1. Background

Oscillatory activities over a wide range of spatial scales, from single neural cells to whole brain regions, are believed to be relevant for brain activities from sensory information processing to consciousness [1]. The phase of low frequency theta oscillations (4–8 Hz) has been often associated with a clock that drives the pyramidal cells and has a crucial role in processing of both spatial and nonspatial information in the hippocampus [2–4] or induces possible long-term potentiation effects [5, 6]. The phase of oscillations is also important in the fast frequency gamma band (30–70 Hz) where event-related phase resettings correlate with visual stimuli [7]. A strong correlation between the phase of theta rhythm and the amplitude of gamma oscillations is also believed to be related to visual stimuli processing and learning [1, 8, 9] and fear-related information processing [10, 11]. In the phase-reset model of cognitive processes [12], the phase of theta rhythm drives the learning.

The phase of oscillations is also used for bridging a much wider frequency range from slow theta rhythms of large neural networks, such as those in the hippocampus, up to the individual fast spiking neurons used for speech decoding [13]. It was found that speech resets background (rest) oscillatory activity in specific frequency domains corresponding to the sampling rates optimal for phonemic and syllabic sampling [13, 14].

The phase of the intrinsic oscillatory rhythm in the suprachiasmatic nucleus of the hypothalamus is constantly reset by light-induced stimuli to produce stable circadian oscillations [15]. The circadian clock generates rhythms and synchronizes them to the environment [16]. The molecular mechanism that generates the circadian rhythm is based on transcription-translation feedback loops [17, 18]. It was found that light pulses phase reset mPER1 gene expression in the suprachiasmatic nucleus [19].

Phase resetting curve method was extensively used for predicting the behavioral response from the activity of large neural networks [20, 21] and also connected with the control theory [22]. PRC was successfully used for controlling the epileptic seizures [23] and Parkinson's tremor [24, 25] and was implemented in neuromorphic circuits to allow real-time seizure prediction and control [26].

If oscillation and phase of oscillation are fundamental concepts and phase resetting is a ubiquitous phenomenon, how can we define unambiguously the concept of phase? How do we define the concept of phase resetting using an unambiguous phase definition?

### 1.1. What Is Phase Resetting?

**Figure 1:**
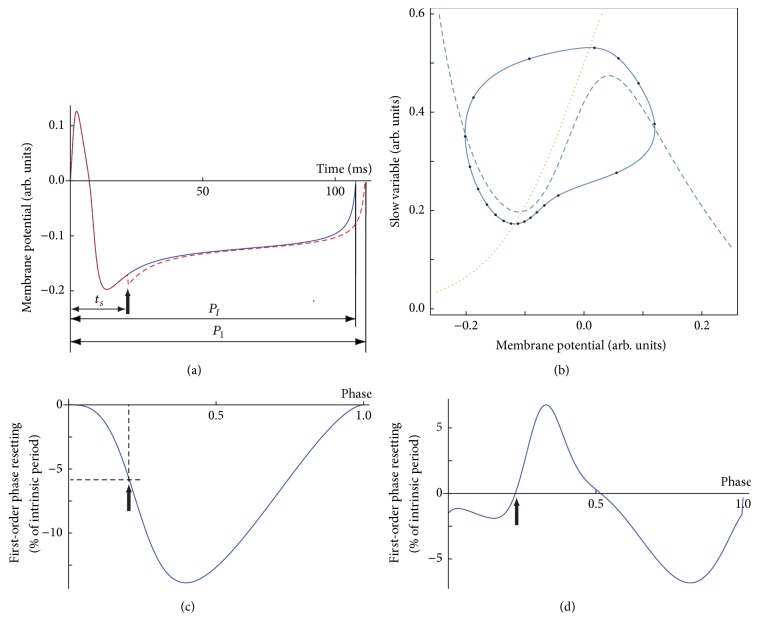
Phase resetting in response to a single inhibitory stimulus. (a) The unperturbed trajectory (continuous line) with an intrinsic firing period *P*_*I*_ is perturbed at a stimulus time *t*_*s*_ measured from an arbitrary voltage threshold (phase reference *φ* = 0). As a result, the new firing period is *P*_1_, which induces a permanent phase shift (resetting) in all subsequent cycles. (b) Phase space portrait of a stable limit cycle. The voltage (dashed) and slow variable (dotted) nullclines show a fixed point that leads to large amplitude, stable, limit cycle oscillations. The 20, equally spaced, solid dots along the limit cycle suggest that the figurative point moves at different speeds through the phase space. (c) A typical type 1 PRC for excitatory perturbations for a class I excitable cell has a unimodal shape. In this case, regardless of the stimulus phase, the next spike is always advanced (negative resetting) (d). For a class II excitable cell, the corresponding type 2 PRC is usually bimodal. The thick vertical arrow shows the timing of a brief current stimulus delivered at *φ* = 0.2.

Although the concepts of phase and phase resetting are used in phenomena spanning wide temporal and spatial scales, from molecular oscillations in the suprachiasmatic nucleus that controls the circadian rhythm to the synchronization of large neural populations, here we only referred to the definition of phase for a single neural oscillator model. A phase resetting curve (PRC) tabulates the transient change in the firing frequency of an oscillator in response to an external stimulus. There are two experimental protocols for measuring the PRC in single, isolated, neurons: (1) single stimulus and (2) repeated (periodic) entrainment. In the case of a single stimulus protocol, a free running neural oscillator with the intrinsic period *P*_*I*_ is perturbed at a certain instant, called stimulus time, *t*_*s*_, measured from an arbitrary phase reference *φ* = 0. As a result of the perturbation, the length of the current cycle that contains the stimulation (see Figure 1(a)) may be transiently shortened or lengthened to a new duration *P*_1_. The relative change of the current cycle duration with respect to the unperturbed duration *P*_*I*_ determines the first-order PRC:

(1) $$\begin{array}{c} F_{1}\left(\;\varphi \;\right)=\frac{P_{1}}{P_{I}}-1. \end{array}$$

A negative value of the PRC means that the next spike was advanced; otherwise it was delayed. Oftentimes, the effect of a stimulus extends to subsequent cycles and is measured by higher order PRCs [27–29]. In the case of repeated period stimuli (entrainment) method, a recurring external stimulus with a fixed period is used [30]. The interpretation of the resetting induced by recurring stimuli and their usage in neural network phase-locked mode prediction is complicated by the facts that (1) the measured resetting is a nonlinear sum of multiple PRC orders and (2) slow currents and/or long-term potentiation/depression may be activated under repeated stimulation.

In addition to the PRCs due to a single stimulus per cycle which is the subject of this study, there are also generalized PRC definitions [31, 32] that attempt to predict the amount of resetting when one neuron receives multiple inputs per cycle; for example, every single neuron in mammalian brains receives thousands of inputs during the same cycle.

The focus of this study is not on how the PRC is interpreted and used for predicting network's activity based on the response of individual neural oscillators (reviews of PRC applications can be found in [27–29]). Here we rather focused on how the phase of an oscillator can be consistently defined and how the PRC can be reliably extracted from data using a consistent and reproducible definition of phase. For this purpose, we only used single stimulus PRCs, although there is nothing in our procedure that changes when recurrent entrainment is used (for a review of generalized PRC method see [31, 32]).

### 1.2. How Is PRC Extracted from Experimental Data?

We assumed that a hyperbolic, attractive, and stable limit cycle describes the neural oscillations [33, 34]. There are at least two widely used definitions of the phase of a neural oscillator. A straightforward technique that is often implemented in experimental electrophysiology uses an arbitrary voltage threshold that is considered as a phase reference *φ* = 0 whenever it is crossed with a positive slope (see Figure 1(a)). At issue is not only the arbitrariness of the voltage threshold reference but, more importantly, the fact that the above definition of phase gives a phase reference value even when the figurative point travels outside the stable limit cycle due to, for example, strong presynaptic perturbations. In such cases, the phase should be undefined (unless a broader definition of phase is adopted based on isochrones; see below). Moreover, since our approach to PRC definition is not limited to “infinitesimal” perturbations, the duration of the stimulus could be quite significant compared to the intrinsic firing period of a neuron. Therefore, the issue of the undefined phase poses a significant conceptual difficulty when deriving phase resetting based on the voltage threshold definition and is exacerbated when attempting to use the PRC for phase-locked mode prediction in neural networks.

If the model equations are known, an alternative approach for phase definition uses the isochrones method [35–37]. Whenever it is possible to reduce the known nonlinear equations of the model to phase equations, the isochrones method allows PRC extraction either directly [35, 37, 38] or by solving the corresponding adjoint problem [39, 40]. The caveat is that the theoretically predicted PRCs are valid very near the bifurcations of periodic firing and only under the assumptions that allow a phase model reduction, usually weak perturbations [41, 42].

For the remainder of this subsection, we used the voltage threshold definition of phase reference due to its simplicity. Voltage traces (see Figure 1(a)) represent one-dimensional projections of stable and attractive limit cycle oscillations (see Figure 1(b)). The PRC can be easily extracted by tabulating the relative change in the firing period of the cycle that contains the perturbation.  Figure 1(c) shows a typical type 1 PRC in response to a brief excitatory rectangular current perturbation (phase advance corresponds to negative resetting). A type 1 PRC looks unimodal and is often associated with class I excitable cells, that is, cells that can produce stable oscillatory activity with arbitrarily low frequency [42–44]. Usually, such excitable cells produce stable oscillations via a saddle-node bifurcation on an invariant circle (SNIC) [45]. A type 2 PRC looks bimodal (see Figure 1(d)) and is often associated with class II excitable cells [42–44]. Class II oscillations emerge through a Hopf bifurcation [45] (see Figure 1(b)). The equally spaced phases along the limit cycle show that phase space speed changes along the limit cycle, which means that perturbations at different phases lead to different recovery speeds and variable phase resettings (see the solid circles in Figure 1(b)). As a side note, it was recently shown that type 1 unimodal PRCs do not always come from a class I excitable cell [46] and that, in fact, all PRCs are bimodal with varying degrees [29, 47].

**Figure 2:**
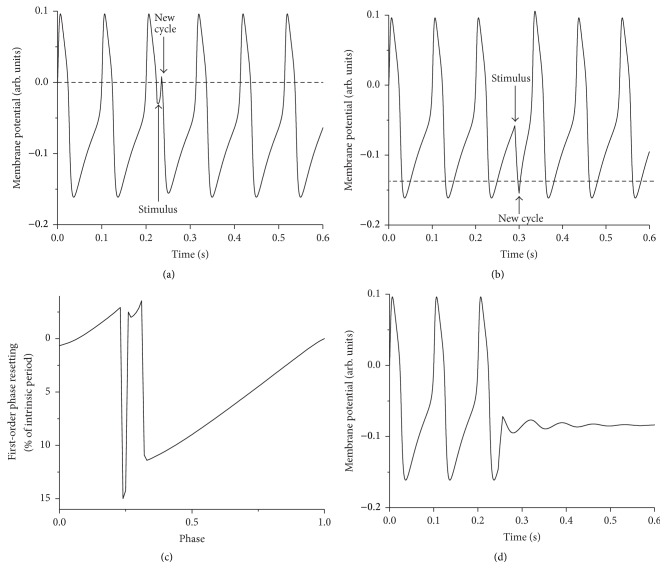
Spurious phase resetting discontinuities. (a) A phase reference defined by an arbitrary voltage threshold (see the horizontal dashed line) counts strong excitatory responses as new cycles. (b) A too low voltage threshold wrongfully counts the response to a strong inhibition as a new cycle. (c) As a result, in both cases the PRC has spurious discontinuities. (d) Additional discontinuities of the PRC are determined by perturbations that abolish the oscillations.

Another obvious shortcoming of the voltage threshold reference for *φ* = 0 phase is revealed when the threshold is not “appropriately” selected. For example, for some voltage thresholds (see dashed line in Figure 2(a)) a relatively large response to an excitatory perturbation may be counted as new cycles, which wrongly suggests a PRC discontinuity (see Figure 2(c)). Moving around the voltage threshold to avoid spurious PRC discontinuities is not the right answer since there will always be perturbations that would cross any arbitrarily selected voltage threshold. For example, a lower threshold may wrongfully count an inhibitory response as a new cycle (see Figure 2(b)). Two additional shortcomings of defining zero phase by a voltage threshold are worth mentioning. First, additional discontinuities of the PRC can occur when a class II excitable cell receives an appropriate stimulus that completely suppresses the oscillations (see Figure 2(d)) [48]. Secondly, in the case of bursting neurons that have a low frequency envelope with superimposed trains of fast spikes, it is particularly challenging to define a phase reference by a voltage threshold. Some external stimuli could easily induce “hard resetting” by moving the figurative point across a phase plane separatrix to a different basin of attraction, which leads to the unsolved question: is what follows the stimulus a new burst (i.e., “hard resetting”) or the continuation of the previous one (i.e., “soft resetting”)? [27, 28].

Although it might be possible for the transiently deformed voltage shape to encode important information regarding the stimulus, we assumed that stimulus characteristics (phase, amplitude, and duration) are only encoded and transmitted to the postsynaptic neurons as permanent phase shifts. Therefore, rather than focusing on the minute changes of the voltage trace shape during the cycle that contains the perturbation, we chose to focus on stable, long-term effects of the stimulus, such as the phase shift of the firing pattern.

### 1.3. Hilbert Transform-Based PRC

Can the PRC be extracted from data without the arbitrariness and artifacts produced by voltage threshold method or the inherent limiting assumptions embedded into the isochrone method?

In this study, we systematically used the Hilbert Transform (HT) approach to compute the PRC from experimental data and showed that the results are independent of the arbitrary and often problematic definition of phase reference. It was previously suggested that HT could offer an objective definition of phase [49, 50] and here we expanded on these ideas and used them to extract the PRC of a single, isolated, neural oscillator.

The Hilbert Transform (HT) is named after David Hilbert (1862–1943), who used it for generating analytical functions in connection with the Riemann problem. The HT is mainly used for extending real functions into analytic functions. Among other useful properties, the HT is bounded on *L*^*p*^ for 1 < *p* < *∞* and it is also bounded on various Sobolev and Lipschitz spaces. In higher dimensions, the HT is used to construct analytic disks with applications in cosmology [51].

Compared to other time-frequency spectral methods, such as short time Fourier transform [52], HT gives sharper frequency and time resolutions [53]. Compared to wavelet and Gabor transforms [54], HT does not require ridge extraction [55, 56]. Compared to Wagner-Ville distribution method [57, 58], which is limited to only linear and stationary data, HT can handle both nonlinearities and nonstationary data. Here we detailed how the amplitude and the phase of HT could be used to extract the PRC from data.

## 2. Model and Method

### 2.1. Model

All numerically generated data used a Morris-Lecar (ML) model neuron [59] that has the advantage of working both as a class I and as a class II excitable cell by changing a small number of parameters [43, 60]. For each excitability class, we used rectangular current pulses with both positive and negative amplitudes to mimic excitatory and inhibitory perturbations, respectively. We only discuss PRCs in response to a single rectangular stimulus applied at a stimulus time *t*_*s*_ measured from an arbitrary reference phase. Unless otherwise stated, the zero voltage crossing with positive slope is the phase reference. As a result of the perturbation, the intrinsic firing period *P*_*I*_ changes to *P*_1_ (see Figure 1(a)). The corresponding phase resetting induced by the stimulus applied at a phase *φ* = *t*_*s*_/*P*_*I*_ is given by (1). In our data, the first two cycles are free runs (no stimulus) only used for determining the intrinsic firing period *P*_*I*_. The perturbation is applied during the third cycle and we recorded at least five subsequent cycles after the perturbation.

### 2.2. Method

The Hilbert Transform (HT) of a time series (membrane potential) *x*(*t*) is defined as [49]

(2) Hxt1πP.V.∫−∞∞xτt−τdτ=1πlimϵ→0+∫t−1/ϵt−ϵxτt−τdτ−∫t+ϵt+1/ϵxτt−τdτ,

where P.V. stands for Cauchy principal value of the improper integral. The analytical signal $\tilde{x}\left(t\right)$ associated with a time series *x*(*t*) is

(3) $$\begin{array}{c} \tilde{x}\left(\;t\;\right)=x\left(\;t\;\right)+iHx\left(\;t\;\right)=A\left(\;t\;\right)e^{i\theta \left(t\right)}, \end{array}$$

where $i=\sqrt{-1}$. The amplitude *A* and the phase *θ* in (3) can be extracted from

(4) A=xt2+Hxt2,θ=arctan⁡Hxtxt.

## 3. Results

### 3.1. The Hilbert Transform

The first step in extracting the PRC from experimental data is performing a HT on the original voltage time series using (2). Although all high level languages (Mathematica, MATLAB, etc.) have predefined functions for HT computation, here we only use MATLAB as a convenient computational tool. For a time series *x*(*t*), the MATLAB code for computing its HT is simply *Hx*(*t*) = hilbert(*x*). The HT has the same amplitude and frequency content as the original sequence and also includes phase information that depends on the phase of the original signal. The HT is useful in calculating instantaneous attributes of a time series, such as its Hilbert amplitude and instantaneous frequency. The instantaneous amplitude is the amplitude of the complex HT (analytical signal) and the instantaneous frequency is the rate of change of the instantaneous phase angle (see (4)).

**Figure 3:**
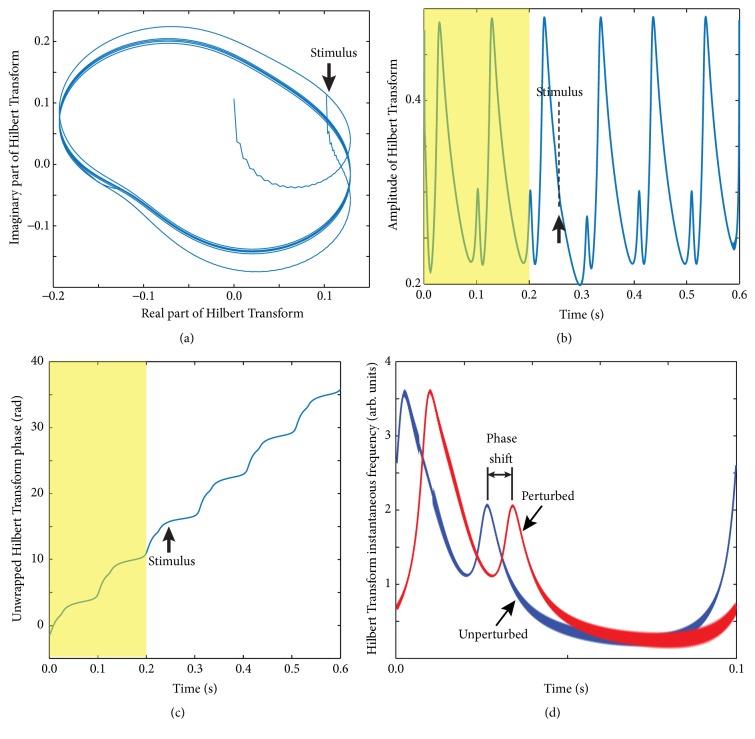
Analytical signal from Hilbert Transform. (a) A typical membrane potential (real part of HT) plot versus its imaginary (phase shifted) part of HT shows a well-defined limit cycle. The arrow indicates the synaptic stimulus that transiently moved the figurative point outside the stable limit cycle. The plot is very similar (besides a phase shift) to the phase space plot shown in Figure 1(b). (b) The amplitude of HT shows the first two unperturbed limit cycles followed by the perturbed cycle and a few recovery cycles. The third cycle that contains the perturbation clearly shows a significant amplitude distortion due to the external stimulus. (c) The unwrapped phase of the analytical signal is also sensitive to external stimuli (although less obvious from the plot). (d) A full period of oscillation of Hilbert frequency (rate of change of Hilbert phase) measured one period before the stimulus (blue trace) clearly shows a permanent phase shift (resetting) when compared against a full period far away from the perturbation (red trace).

The very first check that the HT gives a well-defined phase is a plot of the membrane potential *x*(*t*) versus its HT, *Hx*(*t*) (see Figure 3(a)), which is very similar (except for a phase shift) to the phase space plot shown in Figure 1.

Based on (4), we extracted from the analytic signal $\tilde{x}$ its amplitude (see Figure 3(b)) and its phase (see Figure 3(c)). The first two cycles are free runs (shaded areas in Figures 3(b) and 3(c)), the third cycle contains the perturbation (thick vertical arrow), and the following cycles allow the neuron to recover from the perturbation. As with all HTs, the beginning (the first cycle) and the end (the last cycle) of the data are distorted due to windowing effects [61, 62] and were dropped from our analysis. A solution often used for correcting HT distortion is signal windowing [52]. Both the amplitude (see Figure 3(b)) and unwrapped phase (see Figure 3(c)) show clear distortions during the third cycle, which contains the perturbation, compared to the second (unperturbed) cycle. The rate of change of phase (instantaneous Hilbert frequency) of the analytical signal clearly indicates a significant distortion due to the external perturbation (see Figure 3(d)).

### 3.2. PRC Extraction Based on Hilbert Amplitude of the Analytic Signal

**Figure 4:**
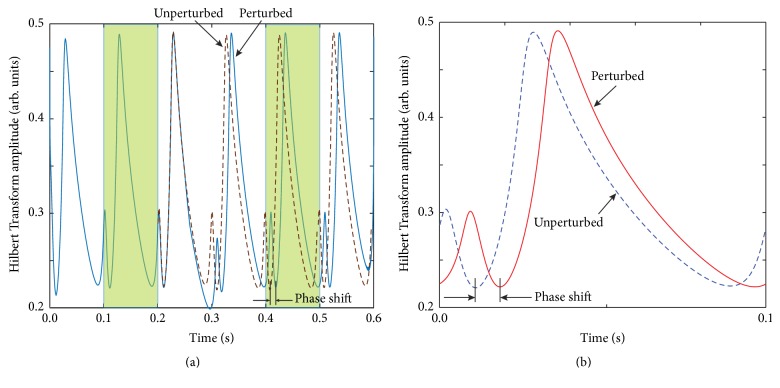
Amplitude of HT analytical signal. (a) The plot of HT amplitude of the first cycle before the perturbation (continuous line) is shifted with respect to a full cycle recorded long after the perturbation dissipates (dashed line). (b) The amount of phase shift (resetting) could be visually estimated by plotting the two cycles together and was automatically computed using the correlation function.

We used Hilbert amplitude profile of the second unperturbed cycle (Figure 3(b)) as a reference pattern to determine the phase shift induced by the perturbation (see shaded area in Figure 4(a) that corresponds to the second, unperturbed cycle).

To determine the phase resetting (permanent phase shift), we compared by how much should Hilbert amplitude trace of a cycle recorded long after the perturbation effect dissipates be circularly shifted to perfectly overlap with the Hilbert amplitude of an unperturbed cycle (see Figure 4(b)). The circular shift we performed is due to the periodicity of the unperturbed signal. Hilbert phase could be used in a similar manner to extract the PRC.

We estimated the amount of phase resetting using (1) the correlation of two amplitude profiles (one before the perturbation and the other one after the perturbation effect dissipates) and (2) by least square minimization between the two selected cycles shaded in Figure 4(a).

How long is long enough for the effect of the perturbation to dissipate? It all depends on how strongly attractive is the limit cycle [39, 43]. For the model parameters we selected, the limit cycle is strongly attractive; that is, the phase space trajectory returns to the unperturbed trajectory after one cycle. Although there are no formal rules and different studies used variable number of cycles to remove the transients [63, 64], in our case a minimum of two cycles after the perturbation suffice.

In order to quantitatively determine the appropriate delay for the perturbation to dissipate, we used the root mean square (rms) of the pre- and poststimulus HT amplitude and frequency profiles as measures of how close the poststimulus profile is to the unperturbed limit cycle. In our simulations, the membrane voltage *x*(*t*) contained two unperturbed cycles that are on the steady limit cycle such that the prestimulus HT amplitude is *a*(*t* + *P*_*I*_), where *t* ∈ (0, *P*_*I*_) and *P*_*I*_ is the intrinsic period of oscillation. We used a 4th-order Runge-Kutta integration method for stiff differential equations and sampled the solution with the sampling time Δ*t*, that is, using *N* = 1000, equally spaced, sampling points per cycle. As a result, the discrete prestimulus Hilbert amplitude profile was *a*(*N* + *k*), with integer index 1 ≤ *k* ≤ *N*. The poststimulus steady amplitude of HT was selected as *a*(delay*∗N* + *k*), where delay ≥ 3 because delay = 2 corresponds to the cycle containing the perturbation (see Figure 3(b)). We defined the rms as

(5) $$\begin{array}{c} a_{\text{rms}}=\sqrt{\frac{1}{n}\overset{n}{\underset{i=1}{\sum }}{\left(\frac{a_{\text{shifted}}\left(\text{delay}*N+i\right)}{a\left(N+i\right)}-1\right)}^{2}}, \end{array}$$

where *a*_shifted_ was the circularly shifted HT amplitude (or frequency) with a certain appropriate delay after the stimulus. Ideally, after the effect of the perturbation dissipated, one should find zero rms of the difference between the pre- and temporarily shifted poststimulus HT amplitude (or frequency) profiles. The rms of pre- and shifted poststimulus profiles served as a selection tool that provided a clear answer as to how long we should wait until the figurative point returned back to the limit cycle. In our numerical simulations, we found that by selecting the poststimulus profile at least two cycles (delay ≥ 3) after the perturbation led to a very low rms that was below 0.1% of the reference amplitude. The small rms did not increase with delay suggesting that we do not need to consider a poststimulus cycle farther than two cycles after the perturbation. The small nonzero rms value comes mostly from the discretization errors.

**Figure 5:**
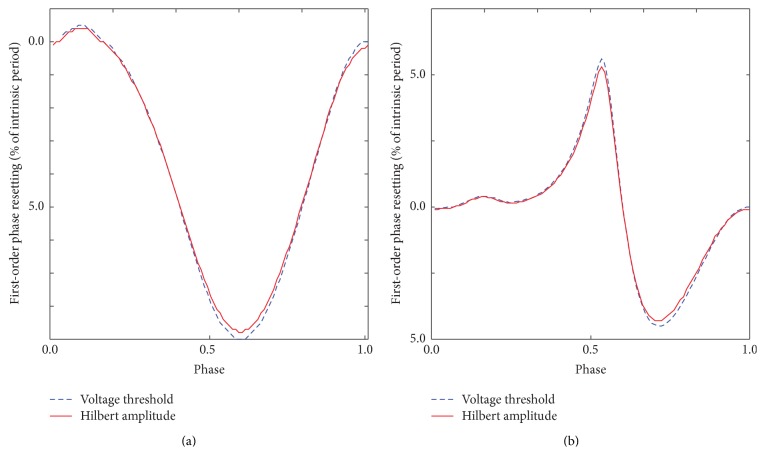
Phase resetting from HT analytic signal. The phase resettings obtained using the arbitrary voltage threshold method (dashed line) and Hilbert amplitude phase shift method (continuous line) are virtually identical both for type 1 unimodal (a) and for type 2 bimodal (b) PRCs.

The resulting PRC from Hilbert amplitude shift method (continuous lines in Figure 5) matches very well the PRC obtained with the traditional method of the arbitrary voltage threshold crossing for both PRC types. Similar results were obtained using Hilbert instantaneous frequency profiles. The HT method does not require a formal definition of zero phase and can be applied to arbitrary stimuli.

## 4. Conclusions

In this paper we found that using either the amplitude or the phase of the HT of membrane potential recorded from electrophysiological data in response external stimulations of neurons is an effective and straightforward approach to extracting phase resetting curves. Compared to the traditional PRC method (see Figure 1) that relies on an arbitrary voltage threshold, extracting the PRC from the analytic signal obtained using HT of membrane potential does not make any assumption other than the fact that the poststimulus membrane potential eventually returns to the unperturbed limit cycle. The closeness of the poststimulus cycle to the unperturbed limit cycle is quantitatively estimated with the rms and can be used to increase the accuracy of the PRC evaluations. As a result, we were able to automatically select the pre- and poststimulus HT amplitude or frequency patterns and find the PRC in real time.

In the presence of noise, the PRC extraction from experimental data is more challenging. For noisy data, there are multiple approaches that have been used and they were selected based on the signal-to-noise ratio. One commonly used measure of noise level is the coefficient of variation (CV) of the interspike interval of the free running neuron. For low CV, that is, low noise level, such as in the case of the experiments carried out by Preyer and Butera [65] on neurons of the abdominal ganglia of* Aplysia californica*, a spline fit of the recorded signal can successfully remove data noise and give the PRC. Others used smoothing with a Gaussian filter [66] or nonlinear regression [67, 68] or approximated the PRC by the solution of a stochastic (noisy) Langevin phase equation [69, 70].
